# Public Health and Epidemiology Informatics

**DOI:** 10.1055/s-0046-1816686

**Published:** 2026-11-03

**Authors:** Brian E. Dixon, John H. Holmes

**Affiliations:** 1 Department of Health Policy & Management, Richard M. Fairbanks School of Public Health, Indiana University, Indianapolis, INUSA; 2 Center for Biomedical Informatics, Regenstrief Institute, Indianapolis, INUSA; 3 Department of Biostatistics, Epidemiology, and Informatics, University of Pennsylvania Perelman School of Medicine, Philadelphia, PAUSA; 4 Institute for Biomedical Informatics, University of Pennsylvania Perelman School of Medicine, Philadelphia, PAUSA

**Keywords:** Public Health Informatics, Epidemiology, Disease Surveillance, Primary Prevention, Secondary Prevention

## Abstract

**Objective:** To identify notable research contributions relevant to public health and epidemiology informatics (PHEI) documented in peer-reviewed articles published in 2024.

**Methods:** A broad search of high-quality biomedical informatics and public health journals was conducted to identify peer-reviewed articles published in 2024 that represent significant contributions to the PHEI discipline and broader field of biomedical and health informatics. The selection process comprised three steps: 1) candidate best papers were first selected by the two section editors; 2) a diverse, international group of external informatics subject matter experts reviewed each candidate best paper; and 3) the final selection of four best papers plus one honorable mention paper was conducted by the editorial committee of the Yearbook. The section editors attempted to balance selection by authors' global region and areas within the PHEI discipline.

**Results:** Selected best papers represent studies that advanced knowledge surrounding the intersection of public health, epidemiology, and informatics methods and applications. In general, papers identified in the search fell into one of the following categories: 1) data management for public health; 2) disease surveillance methods; and 3) infrastructure to support 1 and 2. The best papers spanned several disease targets, including diabetes, influenza-like illnesses, hepatitis C, and COVID. These papers also addressed specialized informatics methods, such as artificial intelligence and machine learning, as well as approaches to data analysis and federated electronic health record systems for public health surveillance.

**Conclusion:** Although the selected papers are notable, there is still much to be done in the domain of informatics that serves epidemiology and public health. This is especially true in epidemiologic surveillance, in which public health authorities seek to rapidly identify and respond to emerging infectious diseases, and the monitoring of chronic, non-communicable diseases such as those identified in the best papers.

## 1. Introduction

Public Health and Epidemiology Informatics (PHEI) integrates methods, knowledge, and theories from the computing and information sciences to support population health and well-being [ 1 ]. Unlike most disciplines within biomedical informatics that focus on applications that diagnose or treat individual patients, PHEI focuses on information systems that target groups or populations of individuals, especially citizens living in the same geography served by a governmental public health authority. The scholarly literature in PHEI is therefore broad and diverse, ranging from population health applications within health care delivery systems (e.g., infection prevention, risk stratification) to governmental public health systems (e.g., disease surveillance) to applications that support health policy (e.g., environmental health, occupational health, social drivers of health) to research into new PHEI methods (e.g., artificial intelligence and machine learning). All areas of public health science benefit from data and information systems, and informatics plays increasingly critical roles in advancing public health practice, policy, and research [ 2 ].

Each year, this section of the *Yearbook* examines recently published scholarly articles on PHEI, identifying trends and selecting the best published papers in the field. From the broader literature on biomedical and health informatics, this section examines papers published in the previous year that represent advancements in PHEI methods and applications. The section highlights novel methods work and original studies that the section editors and peer reviewers believe represent significant contributions to the PHEI discipline and broader field of biomedical and health informatics.

This synopsis paper summarizes the best papers in PHEI published during 2024 and the methods used to identify and select them. Because the theme for the 2025 *Yearbook in Medical Informatics* is “Digital Health and Climate Change”, we especially sought out papers at the intersection of environmental health and informatics. This synopsis and the selected best papers are designed to complement the invited review article by Torres Santos-Olmo *et al.* [ 3 ], which more comprehensively examines recent scholarly work at the intersection of climate health and informatics. Together, along with other contributed papers to the Yearbook, these papers all represent a current look at PHEI and climate health informatics, calling out high quality work and providing a guidepost for trainees and scholars to use in advancing the discipline as well as the impact of informatics on population health.

## 2. Methods

One section editor (BED) performed a literature search using PubMed in January 2025. The query was developed to broadly search the biomedical literature for public health and epidemiology papers relevant to informatics. Both controlled vocabulary terms (e.g., MeSH) and text words were used. Because epidemiology and public health are relevant to many biomedical areas, we explicitly included terms like “surveillance” and “outbreak” in addition to the root terms.

A restricted list of journals was used to constrain the search after it was found that not restricting based on journal led to an unmanageable number of irrelevant articles. Included journals were selected from the first and second quartiles (Q1 and Q2) of ranked journals listed in the 2024 Journal Citation Reports (JCR) from Clarivate for the categories “Medical Informatics” and “Public, Environmental, & Occupational Health”.

Also, because many PHEI applications involve the use of advanced analytics, including artificial intelligence and machine learning, we included “artificial intelligence” as an informatics search term. We employed Boolean logic to identify articles published in English language between January 1, 2024 and December 31, 2024, that contained at least one informatics-related term and one domain-related term. The full query is included as Appendix A.

Information retrieval yielded 661 candidate articles, even with the restriction to specific journals. Using the Covidence systematic review software (Veritas Health Innovation, Melbourne, Australia), the editors performed initial screening of titles and abstracts. Screening removed 512 studies that did not pertain to the theme. Both editors reviewed the remaining articles (N=87) and categorized them into three groups (accept, discuss, and discard) based on their innovativeness, scientific and/or practical impact, and methodological quality. Reasons for exclusion included descriptive review articles or commentary/editorial; published prior to 2024; lacking peer review (published as preprint); the contribution of informatics methods or public health domain was unclear; or the article was focused on system development. The editors tried to focus on including primary studies where a system was deployed or evaluated, or where a novel method was developed with the intention of implementing it in a population health context.

Several candidate articles required discussion amongst the section editors. Where possible, the editors leaned towards selecting candidate papers that focused on environmental and climate health given the theme of the *Yearbook* .

This process yielded 11 articles as candidate best papers. In accordance with the International Medical Informatics Association (IMIA) Yearbook selection process, the candidate best papers were further evaluated and scored by the two section editors, the chief editors of the Yearbook, and invited external reviewers (at least four reviewers per paper) with expertise in medical and/or public health informatics.

## 3. Results

The search yielded a final corpus of 87 PHEI candidate papers for consideration. The PRISMA diagram illustrating the successive yields from applying the search strategy and subsequent assessments for quality and relevance is shown in Appendix B. Several of these papers were commentaries or perspective articles that called for action, encouraging or outlining how informatics methods could be used to support the practice of public health. With one exception which is included here as an “honorable mention”, these articles were excluded from consideration as best papers. Instead, our focus was on those papers detailing the development, implementation, and/or evaluation of informatics solutions that aimed to support public health or epidemiology. Although candidate papers did not have to propose a novel informatics approach, system, or application, they had to be relevant to the biomedical informatics field.

A topic model analysis, using Latent Dirichlet Allocation, was performed on the corpus of 87 candidate papers. The top 10 topics are shown in Table 1.

The papers selected by the section editors in consultation with the editorial board as best papers are summarized in Table 2. Final selection was based on these criteria: 1) reviewer ratings and comments; 2) equity across authors' nation and world regions; and 3) content balance across topic areas within the PHEI discipline. Some topics had more candidate papers than others (as noted in the topic model analysis above), and the editors felt that the final set of best papers should represent the broader discipline of PHEI. A summary of the selected best papers can be found in Appendix C of this synopsis.

## 4. Discussion

There were many good papers in the full corpus of candidate papers, making it challenging for the section editors to narrow down to 11 candidate papers for consideration by external reviewers and the chief editors. Most articles focused on disease surveillance, a foundational aspect of public health. Several papers included descriptions of surveillance systems, risk-prediction models, or the evaluation of systems designed to enhance the detection of disease. These studies focused on a range of diseases, including mpox, HIV, and COVID. One of the best papers by Aronis *et al.* [ 4 ] focused on novel methods for detecting influenza-like illness (ILI). Yet not all of the surveillance papers focused on infectious diseases. Several papers, including those by Conderino *et al.* [ 5 ], Hohman *et al.* [ 6 ], Nghiem *et al.* [ 7 ], and Kraus *et al.* [ 8 ], examined methods or systems designed to enhance surveillance of non-communicable (chronic) diseases such as diabetes and hypertension. The section editors liked the paper by Conderino *et al.* [ 5 ], which detailed rigorous approaches to identifying and assessing information bias in the electronic health record (EHR) data that are increasingly used by health departments for surveillance of infectious and chronic diseases. The section editors also liked the paper by Kraus *et al.* [ 8 ] because it focused on the governance of using EHR data for chronic disease surveillance, which is often not mandatory for providers to report unlike infectious diseases.

Several papers leveraged artificial intelligence (AI) methods, including machine learning, to support surveillance of disease. For example, a candidate paper by de Souza Leandro *et al.* [ 9 ] leveraged ensemble approaches with a variety of data (entomological, epidemiological, demographic and environmental) to examine the transmission of dengue fever in an endemic city. Another candidate paper by Sebastianelli *et al.* [ 10 ] also used ensemble approaches to forecast dengue outbreaks. One of the final best papers by McMurry *et al.* [ 11 ] incorporated AI methods into a system that analyzed EHR data from a federated network using FHIR standards to enhance surveillance. Finally, the paper by Moccia *et al.* [ 12 ] describes methodological considerations for employing machine learning to infer population parameters that are often used by epidemiologists in surveillance applications.

In addition to detection of disease, public health agencies must communicate with lay audiences to promote healthy behaviors, educate people about risks, and disseminate information on the burden of disease within a community. Several papers examined communication methods and approaches. One candidate paper by Slavik *et al.* [ 13 ] examined the use of social media to communicate with lay audiences about the risks of wildfire smoke during events in the U.S. Pacific Northwest. Another candidate paper by Pascual-Ferrá *et al.* [ 14 ] examined techniques for combating misinformation using videos that could be communicated via social media platforms. The best paper in this group by Ishizumi *et al.* [ 15 ] did not examine a specific intervention, instead describing a practical framework that could be used by public health agencies to respond to the threat of misinformation and infodemics.

Another group of papers focused on the modernization of information systems in health systems. These papers largely described the development, implementation, or migration of information systems in support of the public health infrastructure for a state, nation, or region. For example, the paper by Adegoke *et al.* [ 16 ] details the adoption of DHIS2 (District Health Information Software) by nations served by WHO AFRO (African Regional Office), which enhanced national and regional infrastructures for public health surveillance. A paper by Varady and Wood [ 17 ] describes the development and use of a system that integrates with an EHR data warehouse to support population health management, including risk stratification. Infrastructure involves people in addition to technology. A paper from Kirkcaldy *et al.* [ 18 ] describes the U.S. CDC Public Health Informatics Fellowship Program (PHIFP), which establishes a pipeline of individuals trained in PHEI who can serve in local, state, and federal public health agencies following a 2-year, on-the-job training program. The best paper in this group from Maquard *et al.* [ 19 ] provides practical guidance for designing the visualization of information for use by citizens or lay persons based on a comparative study of different approaches that are found in use by health systems. Although all the papers in this group touch different aspects of the public health system, they all impact the infrastructure used by patients, providers, or public health authorities to support health and well-being.

### Limitations

There were many strong PHEI papers published in 2024. The section editors struggled to narrow the candidate list to a final set presented to external reviewers and the chief editors for consideration. Exclusion from the list does not imply the section editors did not find value in a given paper or that there were not another three dozen papers that could have been selected as candidates for best paper.

The search strategy relied on selected top quartile journals included in the JCR. The section editors therefore did not review strong publications outside of core informatics and public health journals. The curated journal list is something the section editors can improve upon in future editions of the *Yearbook* as this was our first time as editors for the PHEI section.

Moreover, the section editors did not use any tools powered by artificial intelligence that are designed to support identification and screening of the published literature. These tools should be explored in the future to facilitate the identification of candidate papers in the growing PHEI literature.

## 5. Conclusions

While the “best papers” on PHEI in 2024 represent a fraction of the strong scientific articles relevant to this topic published before and after this synopsis, included papers represent a broad sampling of papers that pertain to informatics and epidemiology. We tried to focus on key aspects of public health Informatics – including surveillance of disease, policy, data management, and infrastructure. Covering all of these areas seems critical in order to recognize the work that is happening not only by informaticians and public health professionals, but also by others who work in allied professions such as health care providers, administrators, and public policy makers. Future iterations of this section will consider these individuals, the work they perform, and the environments and organizations in which they perform it, casting a wider net to be as inclusive as possible in order to return the most highly relevant documents with the broadest coverage, while still remaining true to the purpose of this section of the IMIA *Yearbook of Medical Informatics* .
